# Impact of Ultra-High-Pressure Homogenisation on the Inactivation of *Bacillus pumilus* and *Bacillus subtilis* Spores in Sheep and Cow Milk

**DOI:** 10.3390/foods13213452

**Published:** 2024-10-29

**Authors:** Anila Antony, Aswathi Soni, Linda M. Samuelsson, Mike Weeks, Meng Wai Woo, Siew-Young Quek, Mohammed Farid, Tanushree Gupta

**Affiliations:** 1 Department of Chemical and Materials Engineering and Department of Food Science, University of Auckland, Private Bag 92019, Auckland 1142, New Zealand; aant520@aucklanduni.ac.nz (A.A.); wai.woo@auckland.ac.nz (M.W.W.); sy.quek@auckland.ac.nz (S.-Y.Q.); 2 Smart Foods and Bioproducts Group, AgResearch Ltd., Private Bag 11008, Palmerston North 4442, New Zealand; aswathi.soni@mpi.govt.nz (A.S.); linda.samuelsson@agresearch.co.nz (L.M.S.); mike.weeks@agresearch.co.nz (M.W.)

**Keywords:** ultra-high-pressure homogenisation (UHPH), bacterial spores, milk processing, novel technologies, *Bacillus*

## Abstract

The efficacy of ultra-high-pressure homogenisation (UHPH) in inactivating *Bacillus pumilus* ATCC 27142 and *Bacillus subtilis* ATCC 6633 spores suspended in sheep and cow milk was investigated. The UHPH treatment was conducted at 200 and 250 MPa with an inlet temperature of 85 °C, resulting in homogenising valve temperatures of 117 °C and 127 °C, respectively. To isolate the role of temperature and pressure in the inactivation of bacterial spores, the UHPH treatment was repeated at 250 MPa with a lower inlet temperature of 70 °C that resulted in a valve temperature of 117 °C. Increasing the pressure and valve temperature resulted in increased inactivation. At 250 MPa with a valve temperature of 127 °C, greater than 5 log CFU/mL reduction was achieved in *B. pumilus* and *B. subtilis* spores in both milk types. Reductions of 0.61 ± 0.03 log CFU/mL and 0.62 ± 0.09 log CFU/mL in *B. pumilus* spores and 1.18 ± 0.04 log CFU/mL and 1.30 ± 0.07 log CFU/mL in *B. subtilis* spores were obtained at 250 MPa with a valve temperature of 117 °C in sheep and cow milk, respectively. The spore inactivation was influenced by both the pressure and temperature, suggesting a synergistic effect, with the latter playing a critical role in the lethality of the treatment. No significant differences in the inactivation of either strain was observed between sheep and cow milk.

## 1. Introduction

Bacterial spore-formers have the ability to form endospores, which provide resistance to stressors like heat and pressure used for milk processing [1]. They can enter the raw milk at the farm level, survive pasteurisation, and potentially germinate under favourable conditions and proliferate in finished products [2]. Aerobic spore formers associated with dairy spoilage predominately belong to *Bacillus* species [3]. Certain psychrophilic strains of *Bacillus* can produce enzymes that break down milk proteins, fat and phospholipids, leading to spoilage of the milk and milk products [4].

Intense thermal treatments such as ultra-high temperature (UHT) (138–145 °C for 1–10 s) have typically been used to inactivate bacterial spores and produce milk that does not contain microorganisms capable of growing at room temperature, which is known as “commercially sterile” milk [5]. However, UHT processing can negatively affect milk quality, taste and appearance [6,7]. UHT is also not suitable for processing milk that is sensitive to heat treatment, such as sheep milk. Due to differences in the micellar structure and protein interactions, sheep milk’s colloidal stability is lower than cow milk’s [8]. The coagulation and sedimentation of mainly protein aggregates have been reported during or after the UHT processing of sheep milk [9,10]. All these factors highlight the need for alternate technologies that can inactivate spores at lower temperatures and extend the shelf life of milk.

Homogenisation is commonly used in the dairy industry to reduce the size of the fat globules (from 1–8 μm to 0.3–0.8 μm) in cow milk before UHT treatment to prevent coalescence during long shelf storage [11,12]. Conventional homogenisers operate with pressures up to 50 MPa and do not contribute to the inactivation of bacterial spores [13]; this is dealt with through the UHT treatment. Ultra-high-pressure homogenisation (UHPH), on the other hand, operates at pressures in the range of 100 to 400 MPa and in addition to homogenising the milk has shown potential for inactivating microorganisms in cow milk [12,14,15,16,17,18]. During UHPH, the fluid food is brought to high pressure (200–400 MPa) in a few seconds using pressure intensifiers and then forced through a narrow valve gap (approximately 2–30 μm) [19,20]. While passing through the valve, the temperature of the liquid increases momentarily due to the shear effects and partial conversion of mechanical energy into heat and pressure build-up (adiabatic heating) [12,21]. The bacterial inactivation observed in the UHPH treatment has been attributed to the resulting pressure and heat that causes intense mechanical forces such as cavitation, turbulence, velocity gradient, impingement and shear stresses [19,22,23].

UHPH has also shown significant potential to inactivate bacterial spores. Previous studies have reported less than 1 log CFU/mL reduction in bacterial spore populations as an effect of treatments at low inlet temperatures (20–50 °C) with homogenisation pressures ranging from 100 to 300 MPa [24,25]. Reverter-Carrión et al. [26] found that increasing the inlet temperature from 20 to 70 °C at 300 MPa increased the inactivation of *Bacillus subtilis* spores from less than 1 log CFU/mL to more than 5 log CFU/mL, highlighting the need for higher inlet temperatures. Furthermore, a study by Amador Espejo et al. [27] evaluated the inactivation potential of UHPH at 300 MPa with inlet temperatures of 55, 65, 75 and 85 °C on *Bacillus cereus, Bacillus licheniformis, Bacillus sporothermodurans, Bacillus coagulans, Geobacillus stearothermophilus,* and *B. subtilis* spores suspended in cow milk. Spore inactivation of at least 5 log CFU/mL was achieved with an inlet temperature of 85 °C and pressure of 300 MPa for all strains tested.

Pasteurisation is the most common heat treatment applied to milk; however, it is unable to inactivate bacterial spores, limiting the shelf life of milk [28]. The significant inactivation of bacterial spores achieved by UHPH demonstrates its potential to extend the shelf life of milk [13]. As a single-step continuous process, UHPH is well suited for application in the dairy industry [19]. The effectiveness of a processing technology must be evaluated in relation to different media, as the sample matrix (i.e., model buffer system, cow or sheep milk) affects how successfully microbial spores can be inactivated [29]. The composition of milk can be highly variable depending on its source [30]. The application and effectiveness of UHPH for the inactivation of bacterial spores suspended in sheep milk have not been reported yet. Even though *Bacillus pumilus* and *B. subtilis* spores have been detected in raw and processed milk, the lethality of UHPH processing on *B. pumilus* spores has not been tested [31,32,33,34,35,36,37,38,39]. This study aims to understand the impact of UHPH at 200 and 250 MPa at high inlet temperatures of 75 °C and 85 °C on the inactivation of *B. subtilis* and *B. pumilus* spores suspended in sheep and cow milk.

## 2. Materials and Methods

### 2.1. Bacterial Strains and Sporulation

#### 2.1.1. Preparation of Spore Suspensions

*B. subtilis* ATCC 6633 was purchased from Fort Richard laboratories (Auckland, New Zealand) and *B. pumilus* ATCC 27142 was obtained from the Institute of Environmental Science and Research (ESR) (Poritua, New Zealand). A wire loop (10 µL) of inoculum from each stock culture was streaked on a Tryptic Soy Agar (TSA) plate (Fort Richard Laboratories) and incubated aerobically at 35 °C for 24 h.

A few *B. pumilus* colonies were transferred into a flask containing 100 mL of Tryptic Soy Broth (TSB) and incubated at 35 °C for 21 days in an orbital shaker incubator at 75 RPM (adapted from Soni et al. [40]). Similarly, a few *B. subtilis* colonies were transferred into a flask containing 100 mL of TSB and placed in a shaking incubator at 35 °C for 24 h. Aliquots of 0.1 mL of this culture were plated on TSA plates and placed in a 35 °C incubator for ten days. The spores were harvested by flooding each TSA plate with 3 mL of sterile cold water and using an L-shaped spreader (Mediray, Auckland, New Zealand) [41]. The bacterial suspension was collected in a sterile glass bottle and placed in an ice bath.

The collected bacterial suspension was treated at 80 °C for 15 min in a water bath preheated to 80 °C and transferred into an ice bath for 10 min to inactivate vegetative cells. The spores were harvested by centrifugation (10,360× *g*, 20 min, 4 °C), the pellets were resuspended in pre-cooled autoclaved deionised water and washed three times by repeating the centrifugation step. The resulting spore suspension had a concentration of 9–10 log CFU/mL and 9–10 log CFU/mL for *B. pumilus* and *B. subtilis,* respectively.

#### 2.1.2. Monitoring Sporulation

Phase contrast microscopy (Motic Microscope BA 410 series, Motic, Richmond, BC, Canada) was used to monitor sporulation as described by Evelyn and Silva [42]. Briefly, the bacterial culture was smeared on a glass slide and heat fixed. The slide was then stained with 5% *w*/*v* malachite green solution (Sigma-Aldrich, St Louis, MI, USA) and steamed for 5 min. The slide was rinsed with deionised water and counterstained with 5% *w*/*v* safranin solution (Sigma-Aldrich).

Sporulation was also monitored by the plate count method as described by Soni et al. [43]. Briefly, the Total Microbial Number (TMN) was determined by plating on TSA plates in duplicate. The Spore Number (SN) was determined by thermally treating 0.5 mL of the suspension in a 1.5 mL sterile Eppendorf tube at 80 °C for 15 min and transferring to an ice bath before diluting and plating. Colony counts for TMN and SN were taken after the plates were incubated at 35 °C for 48 h in aerobic conditions in an incubator preset at 35 °C and cross-checked after 72 h to account for any slow growth. The difference between TMN and SN was used as a proxy to assess sporulation.

### 2.2. Determination of Spore Inactivation

For each experiment, 1 mL of the *B. pumilus* and *B. subtilis* spore suspension was separately inoculated into 1 L of cow or sheep milk and mixed well. The final spore concentration was found to be 6–7 log CFU/mL and 6–7 log CFU/mL for *B. pumilus* and *B. subtilis*, respectively, in each type of milk.

The TMN and SN of the inoculated milk were determined (as described in Section 2.1.2) to ensure that the milk was only inoculated with spores. Samples before and after treatment were enumerated by making serial dilutions in 0.1% peptone and plating them on TSA plates in duplicate. The plate count was taken after incubation at 35 °C for 48 h. The spore inactivation observed in various treatments was calculated using Equation (1), where $N_{0}$ is the concentration of spores before treatment in CFU/mL and $N_{t}$ is the concentration of spores after treatment in CFU/mL. The reduction in spore concentration was reported in log CFU/mL.

Spore Inactivation = log_10_(N_0_/N_t_), (1)

### 2.3. UHPH Unit and Treatment

**Figure 1:**
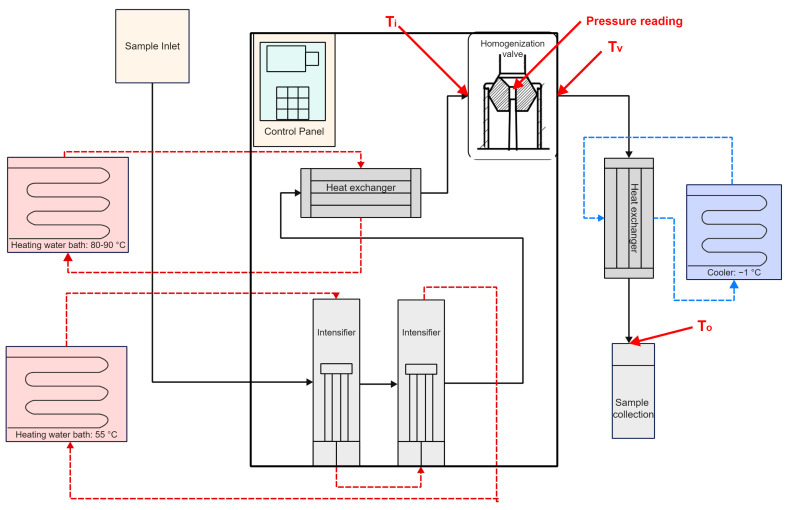
The schematic of UHPH machine where T_i_ is the temperature of milk entering the homogenisation valve, T_v_ is the homogenisation valve temperature, and T_o_ is the temperature of the collected milk.

The UHPH treatments used a pilot scale unit (FPG7575:S6300, Stansted Power Fluid Ltd., Essex, UK) (Figure 1). The equipment consists of two intensifiers driven by a hydraulic pump and high-pressure ceramic valves. This homogenisation valve can sustain up to 400 MPa. The intensifiers are jacketed for hot/cold water circulation to achieve and maintain various inlet temperatures. A spiral-type heat exchanger is fitted between the intensifiers and the homogenisation valve to preheat the sample. Another spiral-type heat exchanger is placed between the valve and the receiving tank to cool the sample rapidly. The inlet temperature before the homogenisation valve (T_i_), the valve temperature (T_v_), the temperature of the collected milk (T_o_) and the operating valve pressures were recorded.

The clean in place (CIP) protocol for the equipment consists of a caustic wash (0.5 M NaOH at 70 °C) for 20 min followed by 5% Oxonia Active (Ecolab Ltd., Hamilton, New Zealand) at 50 °C for 20 min (based on manufacturer’s instructions as well as preliminary trials). Sterile DI water was run through the equipment, collected, and plated to check for microbial growth.

### 2.4. Media/Food Matrix

Pasteurised sheep milk was obtained from Fernglen Farm, Masterton, New Zealand and UHT cow milk (brand not disclosed) from a local supermarket for the experiments.

### 2.5. Statistical Analysis

The concentration of spores (log CFU/mL) is represented as the mean ± SD from four replicates of each experiment. The four replicates of each experiment were conducted using the same batch of milk and the same spore suspension to reduce variability. Statistical analysis of the data was performed using one-way or two-way ANOVA and Tukey’s multiple comparison test in GraphPad Prism 10.3.1 (GraphPad Software, San Diego, CA, USA). Differences of *p* < 0.05 were considered statistically significant.

## 3. Results and Discussion

### 3.1. Temperature-Pressure Control

While passing through the homogenisation valve, the temperature of the liquid increased momentarily due to the shear effects, turbulence, cavitation and the partial conversion of mechanical energy into heat due to pressure build-up (adiabatic heating) [12,21,29,44]. The residence time of the milk at the valve temperature was estimated to be around one second given the flow rate and path through the equipment from the valve to the cooling heat exchanger. Previous studies have estimated the residence time at valve temperature to be 0.2 to 0.7 s [17,27,45]. The estimated residence time is slightly longer in the current study (one second) due to differences in UHPH equipment (flow rate, and pipe length between homogenisation valve and cooling heat exchanger).

Table 1 shows the pressure and temperature variation during the UHPH treatment where T_i_ is the temperature of the milk entering the homogenisation valve, T_v_ is the temperature of the milk in the homogenisation valve, and T_o_ is the temperature of the milk leaving the UHPH machine (Figure 1). Despite the differences in milk types and composition, no differences were observed in the pressure, T_i_, T_v_, and T_o_ achieved by sheep milk and cow milk at the various treatment conditions. Temperature increases of 14–18 °C were observed per 100 MPa. Previous studies using the Stansted pilot scale units have found similar temperature increases [21,45,46,47]. With an inlet temperature of 84 °C, a valve temperature of 117 °C and 127 °C was achieved at 200 MPa and 250 MPa, respectively. When the inlet temperature of milk was lowered to 71 °C, a valve temperature of 117 °C was achieved at 250 MPa. To achieve a valve temperature of 127 °C at 200 MPa, an inlet temperature of 95 °C would be required. As water is used as the heating medium for the heat exchanger that controls the inlet temperature, the maximum inlet temperature that could be achieved was 85 °C. Thus, there are no treatments at 200 MPa that can achieve a valve temperature of 127 °C with the UHPH unit used in the current study. For simplicity, the processing parameters are written in the format “Pressure (MPa)/Valve temperature (°C)” to indicate the pressure and the valve temperature achieved at that pressure.

### 3.2. Spore Inactivation

#### 3.2.1. Impact of Pressure and Temperature on Spore Inactivation

**Figure 2:**
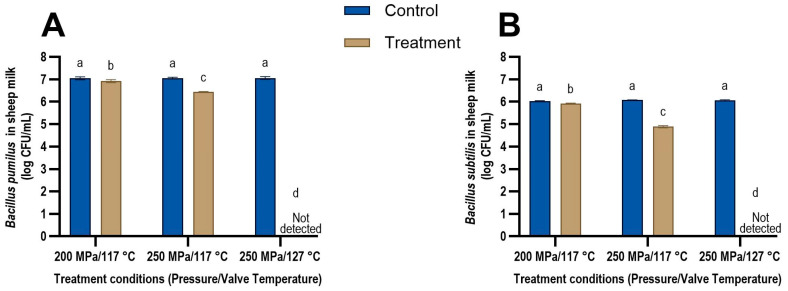
Concentrations of (**A**) *Bacillus pumilus* and (**B**) *Bacillus subtilis* spores in sheep milk (different letters above each bar represent significant differences (*p* < 0.05) before (control) and after (treatment) ultra-high-pressure homogenisation treatment. The limit of detection of the bacterial spores in the milk was 1 log CFU/mL.

**Figure 3:**
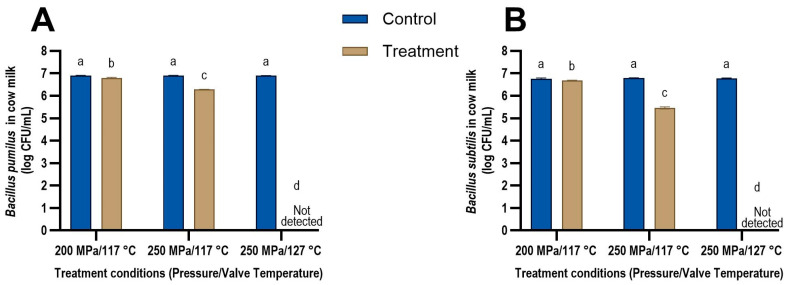
Concentrations of (**A**) *Bacillus pumilus* and (**B**) *Bacillus subtilis* spores in cow milk (different letters above each bar represent significant differences (*p* < 0.05) before (control) and after (treatment) ultra-high-pressure homogenisation treatment. The limit of detection of the bacterial spores in the milk was 1 log CFU/mL.

The impact of UHPH treatment on the spores suspended in sheep and cow milk are presented in Figure 2 and Figure 3, respectively. Pressure and valve temperature were found to be the most important parameters that impact spore inactivation. An increase in pressure and valve temperature led to increased inactivation of both *B. pumilus* and *B. subtilis* spores in both milk types (Figure 2 and Figure 3). These results are in agreement with previous studies that reported an increase in pressure and valve temperatures leading to higher bacterial spore inactivation in phosphate buffer saline (PBS) and cow milk [13,26,27,46].

At 250 MPa and a valve temperature of 127 °C, no bacteria were detected in either milk after treatment and a reduction of at least 5 log CFU/mL was observed for both *B. subtilis* and *B. pumilus* (Figure 2 and Figure 3). Amador Espejo et al. [27] studied the log reduction in *B. subtilis* spores suspended in cow milk at 300 MPa and found only a 0.7 log CFU/mL reduction at a valve temperature of 121 °C. Increasing the valve temperature to 130 °C resulted in a 4.8 log CFU/mL reduction in *B. subtilis* spores, which was similar to the inactivation seen in this study. Conversely, Reverter-Carrión et al. [26] observed a 5 log CFU/mL reduction in *B. subtilis* spores suspended in PBS at 300 MPa and a valve temperature of only 113 °C. The higher inactivation at a lower valve temperature observed by Reverter-Carrión et al. [26] could be due to the differences in the medium the spores were suspended in (PBS vs. cow milk) and the strain of *B. subtilis* used in the experiment. To the best of our knowledge, the inactivation of *B. pumilus* spores during UHPH processing has not been reported previously.

The synergistic effect of pressure and temperature has been hypothesised to cause the bacterial inactivation observed, but the exact mechanism that causes the inactivation has not been elucidated [14,17,26,48]. As the rise in valve temperature is a function of the applied pressure, it is difficult to isolate the impact of temperature or pressure alone on the spore inactivation [13,27].

Two studies modelled the thermal inactivation kinetics of *G. stearothermophilus*, *B. subtilis,* [46] and *Bacillus amyloliquefaciens* [13] spores and compared it to the inactivation observed in UHPH treatment. Both studies found the modelled thermal inactivation to be similar to the inactivation observed in the UHPH treatments, suggesting that the valve temperature may be the key driver for the bacterial spore inactivation in UHPH. Our results support this finding, as increasing the valve temperature from 117 to 127 °C at 250 MPa resulted in the highest inactivation of the bacterial spores (>5 log CFU/mL reduction). In both milk types, the reduction in the number of *B. pumilus* spores was higher at 250 MPa/127 °C (>5 log CFU/mL) than at 250 MPa/117 °C (0.61 log CFU/mL). Similarly, the reduction in the number of *B. subtilis* spores was higher at 250 MPa/127 °C (>5 log CFU/mL) than at 250 MPa/117 °C (1.24 log CFU/mL) in both milk types.

The reduction in the number of *B. pumilus* spores increased from 0.12 log CFU/mL at 200 MPa/117 °C to 0.61 log CFU/mL at 250 MPa/117 °C, while the reduction in the number of *B. subtilis* spores increased from 0.10 log CFU/mL at 200 MPa/117 °C to 1.24 log CFU/mL at 250 MPa/117 °C in both milk types. Thus, a significantly higher spore inactivation was observed when the pressure was increased from 200 to 250 MPa while maintaining a valve temperature of 117 °C (*p* < 0.001) for both species of *Bacillus* in both milk types. These results suggest that the inactivation seen at higher valve temperatures is aided by pressure and the resulting mechanical forces, such as shear stresses, cavitation, and impingement, and they potentially have a synergistic effect. Although the mechanical forces in the homogenisation valve could not be measured in the current study, it is known that increasing pressure increases these forces, which may have contributed to the increased lethality observed at 250 MPa [20,22,49,50]. These results are in agreement with those of Pinho et al. [25], Amador Espejo et al. [27] and Reverter-Carrión et al. [26], who also demonstrated a synergistic effect between valve temperature and pressure.

#### 3.2.2. Effect of *Bacillus* Species on Spore Inactivation

At 250 MPa/117 °C, *B. pumilus* spores had a reduction of 0.61 ± 0.03 log CFU/mL and 0.62 ± 0.09 log CFU/mL in sheep and cow milk, respectively. UHPH treatment at the same pressure and temperature showed a reduction of 1.18 ± 0.04 log CFU/mL and 1.30 ± 0.07 log CFU/mL of *B. subtilis* spores in sheep and cow milk, respectively. These results may suggest that *B. pumilus* spores are more resistant to UHPH treatment than *B. subtilis* spores at 250 MPa/117 °C. Previous studies have also found that the lethality of the UHPH treatment varied among *Bacillus* species suspended in cow milk [46,51]. At 300 MPa/121 °C, a reduction of 4.8, 4.4, 2.6, 2.3, 2.2, and 0.7 log CFU/mL was observed in *B. coagulans*, *B. cereus*, *B. lichenformis*, *G. stearothermophilus*, *B. sporothermodurans*, and *B. subtilis*, respectively. At 300 MPa/139 °C, a reduction in the range of 6.3 to 6.9 log CFU/mL was observed in *B. coagulans*, *B. cereus*, *B. lichenformis*, and *B. sporothermodurans*, while *G. stearothermophilus* and *B. subtilis* only showed a reduction of 5.3 and 5.2 log CFU/mL [51]. In another study, *G. stearothermophilus* and *B. subtilis* were suspended in PBS buffer and treated at 350 MPa/145 °C [46]. The results showed a 5 log CFU/mL reduction in *B. subtilis* but only a 2 log CFU/mL reduction in *G. stearothermophilus* despite the higher pressure and temperature. This difference in the effectiveness of UHPH may be attributed to the different strains of *G. stearothermophilus* tested by the two studies (CECT 47 by Amador-Espejo et al. [27] vs. ATCC 7953 by Georget et al. [46]). These results suggest that the type of *Bacillus* species as well as the specific strain tested impact the results.

#### 3.2.3. Effect of Milk Type on Bacterial Spore Inactivation

No significant difference in inactivation was observed for *B. pumilus* and *B. subtilis* spores suspended in sheep milk compared to *B. pumilus* and *B. subtilis* spores suspended in cow milk. Milk fat has previously been reported to have a protective effect on microbial inactivation during thermal processing [52,53,54]. The fat content of the cow and sheep milk used in this study was reported to be 3.5 and 7 g/100 mL, respectively, by the milk manufacturers’ product label. Sheep milk fat, particularly, has been shown to protect Gram-negative bacteria and *Listeria* species compared to cow and goat milk fat [54]. The protective effect was not just dependent on the higher fat content of sheep milk, as homologous cow fat artificially reconstituted to 10% in cow milk to match sheep milk’s fat content did not exhibit the lower level of inactivation seen in sheep milk.

The influence of milk fat on microbial inactivation during UHPH processing is not well understood. Kheadr et al. [55] reported a higher log reduction in total bacterial count (TBC) in skim milk than whole milk when treated at 200 MPa with an inlet temperature of 28 °C for five passes of UHPH treatment. Conversely, in the same study, a higher inactivation of *Listeria innocua* was observed in whole milk than in skim milk. Another study reported a higher inactivation of *Escherichia coli* O157:H7 in whole milk compared to skim milk [18]. Similarly, Roig-Sagués et al. [47] found that increasing the fat in milk from 0.3 to 15% increased the lethality of UHPH processing against *Listeria monocytogenes*. In the only study that could be found comparing the effectiveness of UHPH in whole and skim milk against bacterial spores, no significant difference was observed between the inactivation of *B. amyloliquefaciens* suspended in whole milk and skim milk [13].

An increased viscosity of the fluid food has been reported to decrease bacterial inactivation in UHPH treatment in model food systems (skim cow milk, soy milk and strawberry–raspberry milk) [56]. Vachon et al. [14] found that UHPH treatment was more effective against *L. monocytogenes* and *E. coli* O157:H7 in PBS than in whole cow milk. In the present work, sheep milk’s higher viscosity and fat content have not shown any protective effects against the inactivation of bacterial spores, as no significant differences between sheep and cow milk were observed across all treatment conditions. Future studies are needed to understand the impact of the UHPH treatment on sheep milk’s temperature-sensitive proteins and assess the shelf-life stability of the UHPH processed sheep milk.

## 4. Conclusions

UHPH treatment at 250 MPa with a valve temperature of 127 °C shows promising results in extending the shelf life of sheep and cow milk, as a greater than 5 log CFU/mL reduction in *B. pumilus* and *B. subtilis* spores was achieved. While the inactivation seems to be primarily driven by valve temperature, pressure and possibly the resulting mechanical forces (such as shear stresses, cavitation, impingement) were found to increase the lethality of the treatment. It is important to test the effectiveness of UHPH treatment across a wide variety of bacterial spores that could be present in milk, as the resistance to treatment among different species varies. UHPH treatment was effective in inactivating spores in both sheep and cow milk despite the differences in the composition of the two milks.

## Figures and Tables

**Table 1:** Temperature and pressure during UHPH treatment.

Target Pressure/Valve Temperature	Pressure (MPa ± SD)	Initial Milk Temperature (°C ± SD)	Inlet Temperature, T_i_ (°C ± SD)	Valve Temperature, T_v_ (°C ± SD)	Outlet Temperature, T_o_ (°C ± SD)
250 MPa/127 °C	257.8 ± 2.9	10.5 ± 1.5	84.6 ± 2.5	127.2 ± 2	19.5 ± 2.3
200 MPa/117 °C	205.0 ± 18.4	9.8 ± 1.3	83.6 ± 1.1	117.3 ± 1.8	17.6 ± 2.5
250 MPa/117 °C	258.0 ± 4.7	10.4 ± 1.4	71.0 ± 4.2	118.3 ± 2.6	17.4 ± 0.5

## Data Availability

The original contributions presented in the study are included in the article, further inquiries can be directed to the corresponding authors.
